# Umbilical cord-derived mesenchymal stem cell extracts ameliorate atopic dermatitis in mice by reducing the T cell responses

**DOI:** 10.1038/s41598-019-42964-7

**Published:** 2019-04-29

**Authors:** Ji-young Song, Hyo Jeong Kang, Hyun Min Ju, Arum Park, Hyojung Park, Joon Seok Hong, Chong Jai Kim, Jae-Yoon Shim, Jinho Yu, Jene Choi

**Affiliations:** 1 Institute for Life Science, University of Ulsan College of Medicine, Asan Medical Center, Seoul, Korea; 2 Department of Pathology, University of Ulsan College of Medicine, Asan Medical Center, Seoul, Korea; 3 Department of Obstetrics and Gynecology, Seoul National University Bundang Hospital, Gyeonggi-do, Korea; 4 Department Obstetrics and Gynecology, University of Ulsan College of Medicine, Seoul, Korea; 5 Department of Pediatrics, Asan Medical Center, University of Ulsan College of Medicine, Seoul, Korea

**Keywords:** Mesenchymal stem cells, Regeneration

## Abstract

Mesenchymal stem cells derived from Wharton’s jelly of the umbilical cord (UC-MSCs) have immunomodulatory properties. The aim of this study was to explore whether extracts of MSCs (MSC-Ex) could augment the low therapeutic efficacy of the whole cells in an *Aspergillus fumigatus* (*Af*)-induced atopic dermatitis (AD) model. LPS- or TNF-α/IFN-γ-stimulated keratinocytes (HaCaT cells) were treated with MSC-Ex, and the *Af*-induced AD model was established in BALB/c mice. In HaCaT cells, MSC-Ex treatment significantly reduced the inflammatory cytokine (IL-6, IL-1β, IL-4, IL-5 and TNF-α), iNOS and NF-κB levels, and upregulated the anti-inflammatory cytokines (IL-10 and TGF-β1). In the AD mice, the MSC-Ex group showed greatly reduced dermatitis, and lower clinical symptom scores and IgE levels. The histological dermatitis scores were also markedly lower in the MSC-Ex-treated animals compared with the MSC-treated group. Decreased levels of IFN-γ (Th1) and IL-17 (Th17), IL-4 and IL-13 (Th2) were detected in T cells and the skin tissue from the MSC-Ex treated AD mice. The therapeutic capacity of MSC-Ex was preserved after lyophilization and reconstitution. MSC-Ex treatment reproducibly suppresses dermatitis and inhibits the induction of inflammatory cytokines in the skin of AD mice. MSC-Ex is therefore a potential new treatment agent for AD.

## Introduction

Atopic dermatitis (AD) is a chronic inflammatory dermatopathy that features eczematous skin lesions with severe pruritus and affects up to 20% of children and 10% of adults^1,2^. The pathogenesis of AD is characterized by dominant type 2 helper T cell (Th2)-mediated abnormal inflammatory responses, resulting in B lymphocyte-mediated increases in serum immunoglobulin E (IgE)^3–5^. Subsequent degranulation of mast cells by the elevated IgE releases and recruits lymphocytes and eosinophils to the AD lesion. The current clinical management of AD comprises topical corticosteroids and systemic immunosuppressive agents^5,6^. However, the long-term use of these drugs is associated with a series of well-documented side effects, including growth disturbance, osteoporosis, cataracts, and the development of lymphopenia^7–9^.

Several recent studies have indicated that the use of mesenchymal stem cells (MSCs) is a promising therapeutic approach for AD. Shin and colleagues reported that intravenously injected human adipose tissue-derived MSCs (AT-MSCs) alleviate AD by suppressing B lymphocyte proliferation and maturation via COX-2 signaling^10^. Moreover, subcutaneously injected human umbilical cord blood-derived MSCs (UCB-MSCs) have shown a dose-dependent clinical efficacy in moderate-to-severe adult AD patients in a clinical trial (phase I/IIa); about 58% of the patients in the trial exhibited a 50% reduction in the Eczema Area and Severity Index score^11^.

Human umbilical cord-MSCs (UC-MSCs) are obtained from the Wharton’s jelly of the umbilical cord and are distinguishable from the MSCs present in UC blood (UCB-MSCs)^12,13^. UC-MSCs have been most widely used for the treatment of various inflammatory diseases because they can be easily isolated in large quantities from postnatal cord tissues^14,15^ and express low levels of type I HLA antigen, but do not express type II HLA antigens or T cell co-activators^16^. Many prior studies have reported that only a limited number of engrafted MSCs reach inflamed damaged tissues and are rapidly cleared post-administration^10,17^. In our current study, we investigated the therapeutic effects of UC-MSC extracts (MSC-Ex) in an *Aspergillus fumigatus* (*Af*)-induced AD mouse model. Treatment with MSC-Ex efficiently blocked the induction of pro-inflammatory cytokines and reduced both atopic histopathological symptoms and immune responses in these animals. The clinical therapeutic effects of MSC-Ex were thus found to be superior to that of MSCs.

## Results

### MSC-Ex suppresses pro-inflammatory cytokines in keratinocytes

**Figure 1:**
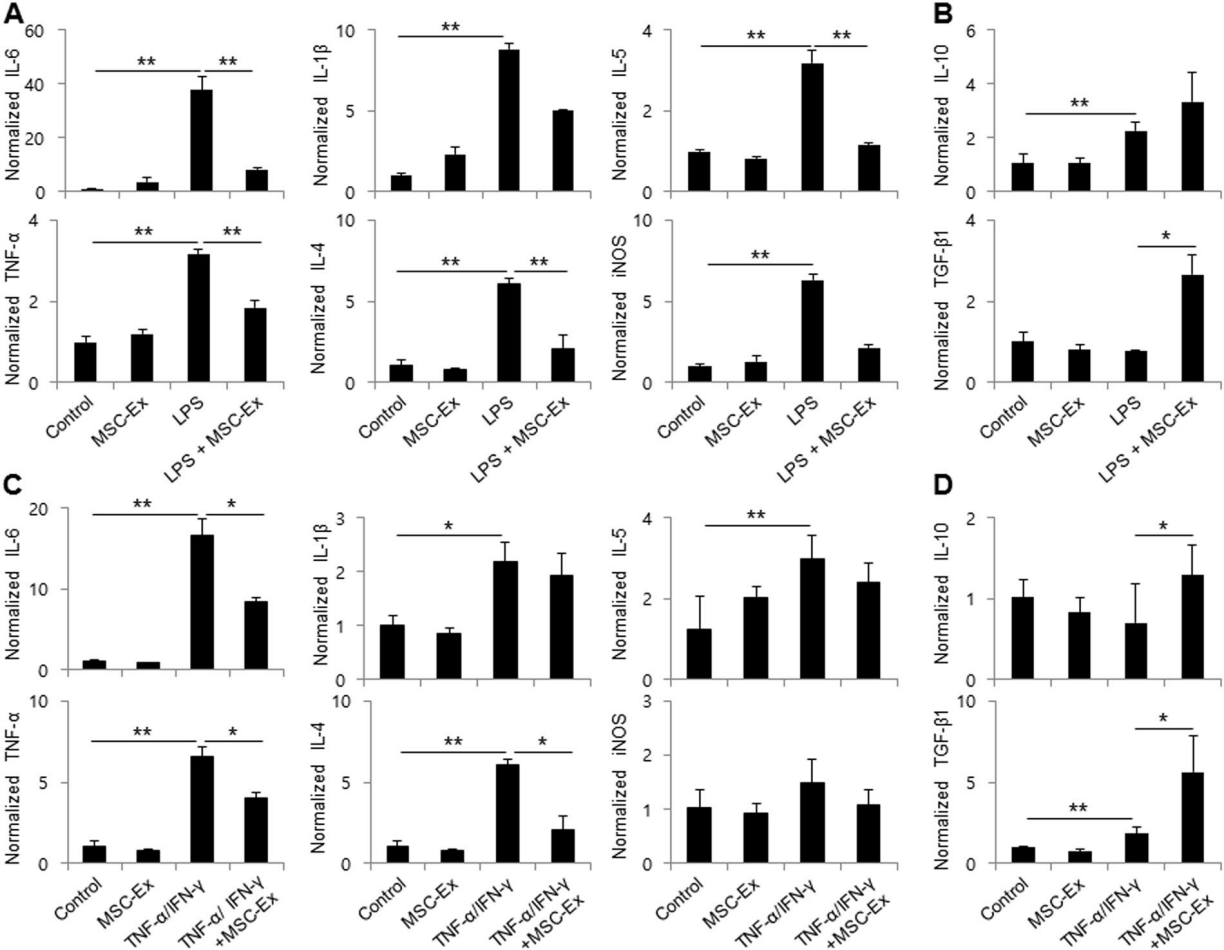
MSC-Ex decreases the expression of pro-inflammatory cytokines in LPS- or TNF-α/IFN-γ-stimulated HaCaT cells. The relative expression levels of the inflammatory-associated genes were evaluated by qRT-PCR after stimulation by LPS for 4 h (100 μg/ml) or TNF-α/IFN-γ (10 ng/ml each) for 2 h and then subsequently after MSC-Ex (30 μg/ml) treatment for 24 h with LPS: IL-6, TNF-α, IL-1β, IL-4, IL-5 and iNOS (**A**,**C**), IL-10 and TGF-β1 (**B**,**D**). Columns represent the means of three independent experiments; bars indicate standard deviations. *P < *0*.*05*, **P < *0*.*005*, ***P < *0*.*001*.

HaCaT cells were stimulated with LPS (100 μg/ml) for 28 h to investigate whether MSC-Ex treatment generated anti-inflammatory effects in human keratinocytes. As expected, LPS increased the mRNA levels of the proinflammatory cytokines IL-6, IL-1β, and TNF-α, as well as those of the Th2 cytokines IL-4 and IL-5. MSC-Ex treatment significantly decreased the mRNA levels of these five cytokines and suppressed the expression of inducible NO synthase (iNOS) in LPS-stimulated HaCaT cells (P < 0.01). In contrast, MSC-Ex treatment increased the mRNA levels of the anti-inflammatory cytokines IL-10 and TGF-β1 (P < 0.05) (Fig. 1A,B). MSC-Ex suppressed the expression of the above-mentioned Th1- and Th2-type cytokines after stimulation with TNF-α and IFN-γ (Fig. 1C,D). These data indicate that MSC-Ex suppresses the Th1 and Th2 inflammatory responses of human keratinocytes.

### MSC-Ex inhibits NF-κB activity

**Figure 2:**
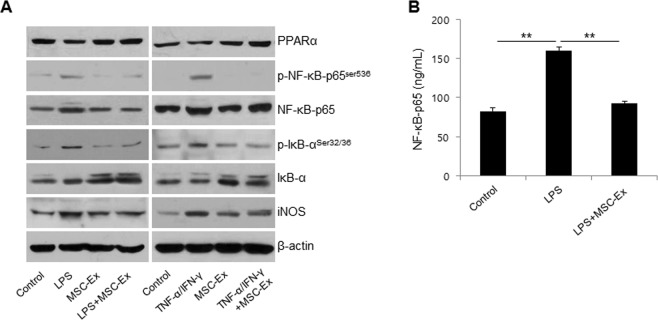
MSC-Ex restores the PPARα activity in HaCaT cells. (**A**) HaCaT cells were treated as described in Fig. 1 and western blot analyses were performed using primary antibodies for PPARα, NF-κB-65, p-NF-κB-p65^Ser536^, IκB, p-IκB^Ser32/36^ and iNOS. β-Actin was used as the loading control. (**B**) The protein levels of NF-κB were determined by ELISA. Columns represent the means of three independent experiments; bars indicate standard deviations.). *P < *0*.*05*, **P < *0*.*005*, ***P < *0*.*001*.

NF-κB plays a key role in regulating the immune response to inflammatory diseases. PPARα inhibits NF-κB-dependent transcriptional activation and subsequent inflammatory responses^18,19^. To further examine the mechanisms underlying the reduction in inflammatory responses by MSC-Ex, we assayed NF-κB and PPARα in LPS- or TNF-α and IFN-γ-stimulated HaCaT cells (Fig. 2A). MSC-Ex significantly reduced the NF-κB and iNOS protein and inversely increased PPARα and IκB, the inhibitor of NF-κB. In addition, the MSC-Ex-treated cells exhibited low levels of p-IκB-α at Ser32/36 and p-NF-κB at Ser536, which are required for the transactivating function of NF-κB, compared with the stimulated control. The NF-κB protein levels following MSC-Ex treatment were confirmed by ELISA (P < 0.01) (Fig. 2B). These data supported that the immune mediator NF-κB is strongly antagonized by MSC-Ex in inflammatory keratinocytes.

### MSC-Ex ameliorates *Aspergillus fumigatus (Af)*-induced AD

**Figure 3:**
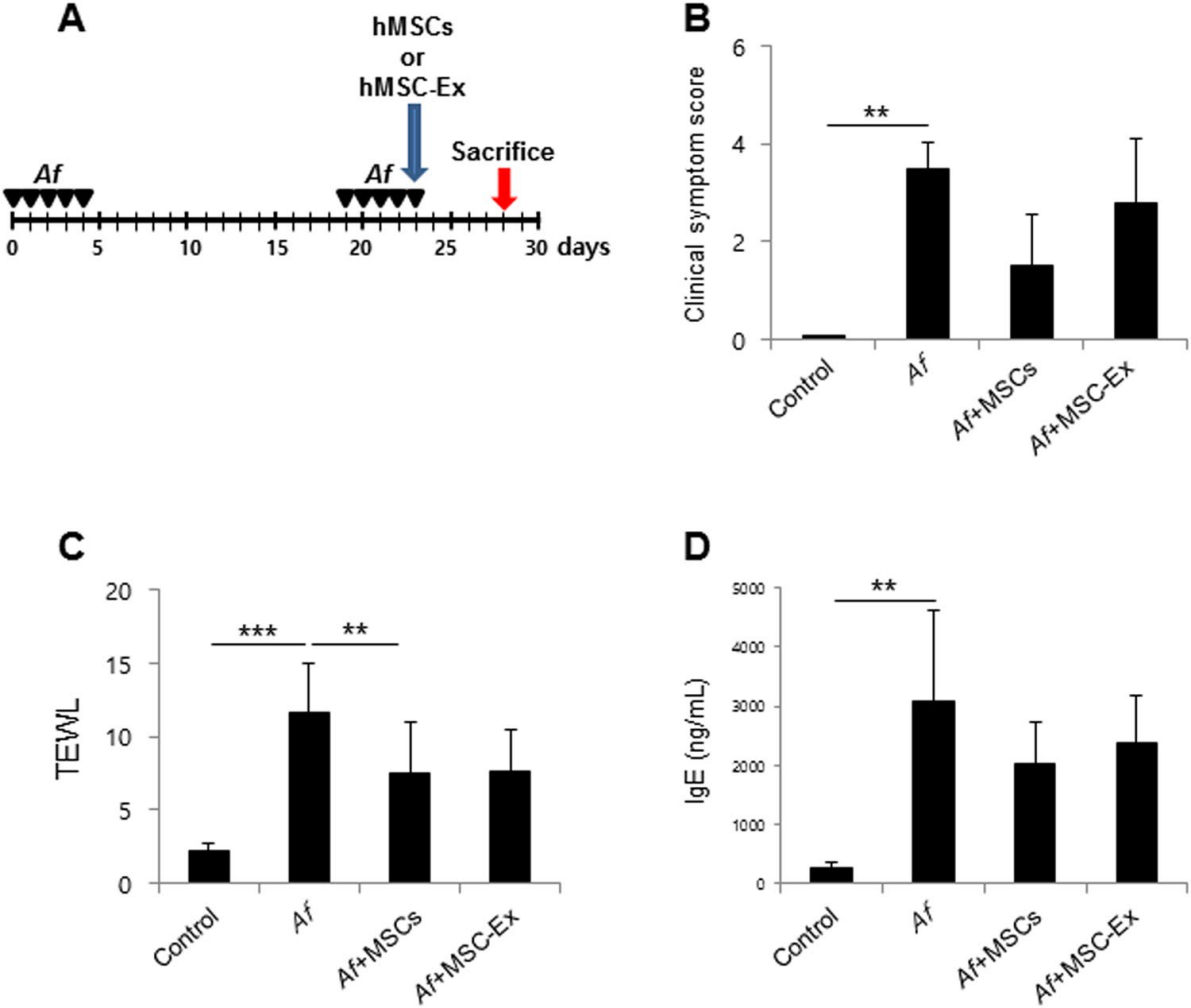
MSC-Ex treatment attenuates AD in the *Af*-induced mouse model. (**A**) Schematic protocol for *Af* -induced mouse AD and s.c. injection of MSCs (2 × 10^6^ cells) or MSC-Ex (300 µg), respectively. (**B**) Clinical symptom scores were calculated on the last day of the experiment (day 28) based on the clinical Psoriasis Area and Severity Index. (**C**) TEWL in the mouse dorsal skin was measured at the end of the experiment. (**D**) The IgE levels in serum were measured using ELISA. Data are represented as the means ± s.d. (n ≥ 6 mice per group). *P < *0*.*05*, **P < *0*.*005*, ***P < *0*.*001*.

We hypothesized that a robust administration of paracrine factors using MSC-Ex may be a viable alternative to UC-MSCs in the treatment of AD as it would overcome the low survival rate of the whole cells *in vivo*. An *Af*-induced AD mouse model was used to investigate the therapeutic effects of MSC-Ex *in vivo*. UC-MSCs (2 × 10^6^ cells/mouse) and MSC-Ex (300 μg) prepared from an identical number of MSCs were administrated subcutaneously to the AD mice on day 23 (Fig. 3A). Both treatments significantly reduced the clinical scores after 5 days. Compared to the MSC-Ex treatment, however, UM-MSCs showed a trend towards a stronger reduction of the clinical symptom scores (Fig. 3B). UC-MSC and MSC-Ex treatments both reduced the TEWL and IgE levels in the serum to a comparable degree (Fig. 3C,D). Taken together, these data indicated that UC-MSC and MSC-Ex have a similar therapeutic efficacy against AD that may result from the systemic regulation of allergic responses.

### MSC-Ex has a superior therapeutic efficacy to UC-MSCs in the AD mouse model

**Figure 4:**
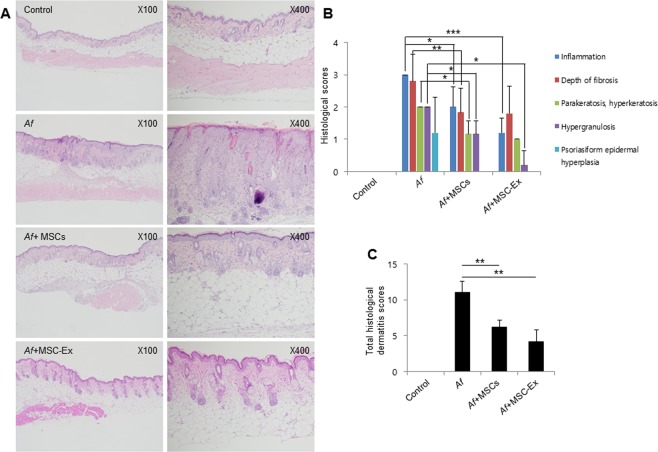
MSC-Ex reduces dermatitis in the AD mouse model. (**A**) Macroscopic appearance of the skin sections of the dorsal surface (original magnification, 100x and 400x). (**B**) Histological symptoms of AD used to assess disease severity by histology. (**C**) Total histological AD scores were determined. Data are represented as the means ± s.d. (n ≥ 6 mice per group). *P < *0*.*05*, **P < *0*.*005*, ***P < *0*.*001*.

We assessed the severity of AD histologically in the mouse model using a scoring system for hyperkeratosis, parakeratosis, psoriasiform epidermal hyperplasia, hypergranulosis, and prominent or absent spongiosis. The MSC-Ex treated group showed significantly improved dermatitis parameters such as lower hypergranulosis and psoriasiform epidermal hyperplasia compared with the animals who received MSCs (Fig. 4A). However, we did not detect differences in the depth of fibrosis between the MSC-Ex-treated and the MSC treated groups (Fig. 4B). Collectively, our findings indicated greatly decreased total histological dermatitis scores in the MSC-Ex treated group (Fig. 4C) and that MSC-Ex had markedly superior therapeutic efficacy to MSCs in the AD mouse model (mean of total score, 6.0 *vs* 4.2).

### MSC- Ex suppresses T cell activation

**Figure 5:**
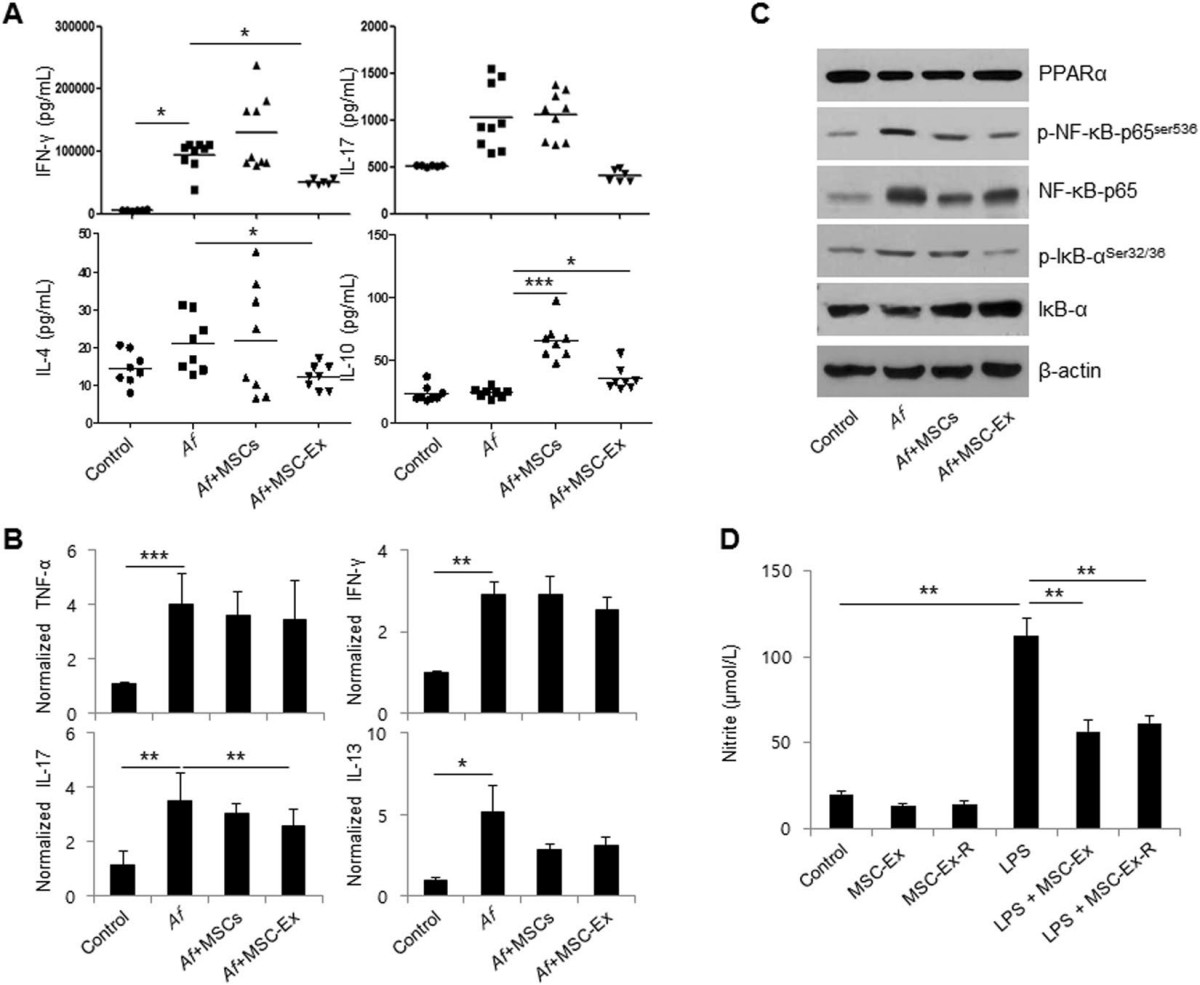
MSC-Ex downregulates LN-derived Th1, Th2 and Th17 cell activation. (**A**) IFN-γ, IL-17, IL-4 and IL-10 levels in anti-CD3/CD28 stimulated lymphocytes from the LNs of *Af*-treated mice were measured by ELISA. Data are represented as the means ± s.d. (n ≥ 6 mice per group). (**B**) The relative expression levels of the inflammatory-associated cytokines, TNF-α, IFN-γ, IL-17 and IL-13, were evaluated by qRT-PCR in the skin tissues of AD mice. (**C**) The protein levels of PPARα, p-NF-κB-p65^Ser536^, NF-κB-65, p-IκB^Ser32/36^ and IκB, were analyzed by immunoblotting in the skin tissue of AD mice. (**D**) The levels of nitrite were measured using the culture media from the basolateral side of MSC-Ex and RAW 264.7 co-cultures after 4 h of LPS (1 μg/ml) treatment and subsequent MSC-Ex (30 μg/ml) administration for 24 h. Columns represent the means of three independent experiments. Bars indicate standard deviations. *P < *0*.*05*, **P < *0*.*005*, ***P < *0*.*001*.

The pathogenesis of AD is defined by an imbalance in Th1, Th2, Th17, and Treg cells^20^. To further investigate T cell differentiation in our AD mice, the levels of IFN-γ (Th1), IL-17 (Th17), IL-4 (Th2) and IL-10 (Th9) were measured in anti-CD3/CD28 activated T cells derived from the local LNs of the UC-MSC- or MSC-Ex-treated animals. The levels of the pro-inflammatory cytokines IFN-γ and IL-17 were significantly decreased in T cells in the MSC-Ex-treated group compared to the untreated control and UC-MSC-treated groups. Moreover, MSC-Ex treatment resulted in levels of IL-4 and IL-10 similar to those of the healthy control group (Fig. 5A). Next, we examined the expression levels of pro-inflammatory cytokines in the skin tissue of AD mice. The expression levels of the pro-inflammatory cytokines TNF-α, IFN-γ, IL-17, and IL-13 were decreased (Fig. 5B), and immunoblotting showed (Fig. 5C) that the activation pattern of the NF-κB pathway was identical to that in HaCaT cells (Fig. 2A). Hence, MSC-Ex promotes immunosuppressive and suppresses T cell activation, and shows a higher therapeutic efficiency than the whole cells.

### The therapeutic capacity of MSC-Ex is preserved after lyophilization

MSC-Ex would have to show product stability to be developed as a future therapeutic agent. We examined whether the stability and performance of MSC-Ex would be preserved after lyophilization. MSC-Ex powder was reconstituted in PBS after 5 days of freeze drying and then administered to LPS-stimulated RAW 264.7 macrophage cells. In the co-cultured RAW 264.7 cells, reconstituted MSC-Ex (MSC-Ex-R) significantly reduced the levels of nitrite, a principal final metabolite of NOS and an inflammation marker. The therapeutic efficiency of this preparation was comparable with that of the original MSC-Ex (Fig. 5D). These data further support the potential for MSC-Ex to be used as a cell-free treatment for AD.

## Discussion

MSC therapy has emerged as a promising strategy for a range of immune-mediated conditions and previous studies have reported encouraging results with these cells in inflammatory diseases. However, the clinical outcomes of these treatments with respect to efficiency are variable, likely due to the heterogeneity resulting from the use of bone marrow-, adipose-, UC blood-, or UC (Wharton’s jelly)-derived MSCs, the homing efficiency of MSCs, and the different microenvironments that the engrafted cells have encountered in different studies. In our current study using an AD mouse model, we tested the proposition that umbilical cord-derived MSC extracts (MSC-Ex) would be as safe but have superior efficacy to MSCs by analyzing their paracrine effects. Our results indicated that subcutaneously injected MSC-Ex significantly improves the inflammatory symptoms in *Af*-induced AD mice and shows no loss of potency after freeze drying and reconstitution in buffer.

One of the ongoing debates in stem cell therapy is the homing efficiency of engrafted MSCs to a site of injury or inflammatory sites. In some preclinical disease models, the infused stem cells have been found to be entrapped mainly in other organs such as the liver and lungs^17^. Recently, Shin and colleagues reported that the majority of MSCs are detected in the lung and heart in a murine AD model after intravenous injection^10^. Our present *in vitro* and *in vivo* data provide evidence that robust administration of anti-inflammatory paracrine factors contained in the MSC-Ex preparation inhibited T-cell-driven inflammatory responses via reductions in the levels of IFN-γ (Th1 cell marker), IL-17 (Th17 cell marker), IL-4, IL-5 and IL-13 (Th2 cell marker) and B-cell-mediated serum IgE (Figs 1, 3 and 5), which were reported in chronic colitis models^15^. These results further indicate that MSC-Ex attenuates allergic responses systemically and has an efficacy via a one-time injection that is comparable to the equivalent number of injected and entrapped MSCs that secrete anti-inflammatory cytokines over 5 days (Fig. 3).

The toxicity of MSCs has been evaluated previously by testing various dose levels. Establishing a single cell dose limit will be ideal for future applications of these cells in clinical therapy. We here injected 2 × 10^6^ UC-MSCs per mouse or MSC-Ex prepared from this same number of cells (equaling 300 μg per mouse). These dosage levels did not cause any adverse events or abnormal inflammatory responses at the injected sites.

A number of studies have reported that MSCs secrete soluble factors, but that they become undetectable only a few days after injection^15,21,22^. The most interesting point in this regard is that the therapeutic effects of MSCs seem to be dependent on the specific microenvironment that the cells encounter after their injection^23^. Polchert *et al*. have reported that bone marrow-isolated MSCs are effective at day 2 and 20, but not at day 0 and 30 in a graft *versus* host disease (GVHD) mouse model^24^. Toll-like receptor 3/4 (TLR3/4)-activated MSCs promote inflammatory responses via the production of inflammatory mediators such as IL-1β, IL-6, IL-8/CXCL8, and CCL5 against pathogens^25^. The contradictory or even opposite outcomes of inflammatory disease models and clinical studies could be due to different cytokine concentrations and differences in the cross talk between MSCs and the local microenvironment. Our current results in the mouse show that MSC-Ex ameliorates the clinical symptoms of AD (dryness, scaling, erosion, excoriation, and hemorrhage) as well as reduces the TEWL and IgE levels, but we found a lower local immunologic response level at the site of MSC-Ex injection compared with that of MSC injection (unpublished data).

Of note, we found that the levels of IL-17 and IFN-γ were greatly reduced in T cells from the MSC-Ex-treated group compared with the MSC-treated group (Fig. 5A). The percentage of Th17 cells was significantly correlated with that of IFN-γ-producing Th1 cells in the peripheral blood of patients with AD and was associated with AD severity^26^. Thus, we contend that MSC-Ex modulates IL-17-secreting Th17 as well as Th1/Th2 cells such that the physiological conditions are shifted from pro-inflammatory to anti-inflammatory. Therefore, MSC-Ex immediately interferes with both the innate and adaptive immune systems, rather than slowly reprograming the immune cells via cell-to-cell interactions in host tissues, as MSCs play a role. We utilized *Aspergillus fumigatus*, a fungal pathogen, in this AD model. Most patients with asthma have elevated serum IgE levels and concomitant atopic eczema and allergic rhinitis. Aspergillus species are strong fungal allergens^27^. We believe that our AD model and MSC-Ex treatment mimic the atopic conditions and the treatment thereof in a clinical setting, and MSC-Ex could provide an alternative therapeutic tool for AD.

## Materials and Methods

### Ethics statement

This work was approved by the Institutional Review Board of Asan Medical Center (authorization no. 2015-0303). The research was conducted in accordance with Helsinki Declaration. All of the participating women provided written informed consent. All animal experiments were performed in accordance with relevant guidelines and regulations of the Animal Ethics Committee of Asan Medical Center (authorization no. 2015-12-220) accredited for laboratory animal care by Ministry of Food and Drug Safety of South Korea.

### Cell culture and treatment

HaCaT (human keratinocyte) cells were maintained in DMEM (Invitrogen-Gibco, Carlsbad, CA) containing 10% fetal bovine serum, penicillin (100 U/ml), and streptomycin (100 g/ml) (Invitrogen-Gibco) at 37 °C in a humidified 5% CO2 incubator. HaCaT cells were pretreated with bacterial LPS (*E. coli*, serotype 055:B5, 100 μg/ml; Sigma-Aldrich, St. Louis, MO) for 4 h or TNF-α (300-01 A; PEPROTECH, Rocky Hill, NJ) and IFN-γ (300-02; PEPROTECH) (10 ng/ml each) for 2 h and then treated with MSC-Ex (30 μg/ml) in the presence of LPS or TNF-α/IFN-γ for an additional 24 h. RAW264.7 cells were incubated with LPS (1 µg/ml) for 4 h. After incubation, the RAW264.7 cells were washed three times in ice-cold UC-MSCs were washed twice with cold phosphate-buffered saline (PBS), and treated with MSC-Ex (30 μg/ml) for 24 h.

### Isolation and expansion of UC-MSCs

UC-MSCs were prepared as previously described^15^. Briefly, after removing the vessels and amnion, Wharton’s jelly tissues were minced and digested for 3 h in MEM (11095-080; Invitrogen-Gibco) with 0.1% collagenase A (10103578001; Roche, Mannheim, Germany) at 37 °C in a shaking incubator. They were then filtered through a 70 μM mesh (103760; BD Falcon, San Jose, CA) and pelleted using low-speed centrifugation at 200 × g for 10 min. The isolated cells were cultured in DMEM supplemented with 10% FBS. MSCs at passages 3–5 were pooled for subsequent MSC-Ex or MSC therapies. UC-MSCs were characterized with a panel of surface markers (positive for CD29, CD73, CD90 and CD105 and negative for CD34 and CD45) (Supplementary Fig. S1).

### Preparation of MSC-Ex

UC-MSCs were washed twice with cold PBS and resuspended in five times the packed cell volume of PBS. After 30 min of incubation on dry ice, the cells were transferred to a water bath maintained at 37 °C for 3 min. The cells then underwent two cycles of freezing and thawing. After centrifugation at 12,000 × *g* for 10 min, the supernatant was collected and stored at −80 °C. For lyophilization, frozen MSC-Ex samples were transferred to a lyophilizer (Alpha 1–4 LSC plus Entry Freeze Dryer; John Morris Group, Queensland, Australia) operating at −55  °C and 0.0715 mbar. The frozen lysates were dried for ~24 h and reconstituted in PBS.

### Quantitative real-time reverse transcription-polymerase chain reaction (qRT-PCR)

Total RNA was isolated using the FavorPrep Total RNA Mini Kit (FABRK001; Vienna, Austria). First-strand cDNA was synthesized from 1 μg of total RNA using the SuperScript^TM^II enzyme (18064-014; Invitrogen). qRT-PCR was performed on an Applied Biosystems 7900HT Fast Real-Time PCR System using the Power SYBR Green PCR Master Mix (326759; Warrington, UK) with the following primers: GAPDH, GAAGGTGAAGGTCGGAGTC (forward) and GAAGATGGTGATGGGATTTC (reverse); IL-6, AATTCGGTACATCCTCGACGG (forward) and GGTTGTTTTCTGCCAGTGCC (reverse); IL-1β, AAAAGCTTGGTGATGTCTGG (forward) and TTTCAACACGCAGGACAGG (reverse); TNF-α, GGAGAAGGGTGACCGACTCA (forward) and CTGCCCAGACTCGGCAA (reverse); IL-4, CCGTAACAGACATCTTTGCTGCC (forward) and GAGTGTCCTTCTCATGGTGGCT (reverse); IL-5, GGAATAGGCACACTGGAGAGTC (forward) and CTCTCCGTCTTTCTTCTCCACAC (reverse); iNOS, TCAGCCAAGCCCTCACCTAC (forward) and CCAATCTCTGCCTATCCGTCTC (reverse); IL-10, GATCCAGTTTTACCTGGAGGAG (forward) and CCTGAGGGTCTTCAGGTTCTC (reverse); TGF-β1, CACCCGCGTGCTAATGG (forward) and TGTGTACTCTGCTTGAACTTGTCAT (reverse); mβ-actin, GGCTGTATTCCCCTCCATCG (forward) and CCAGTTGGTAACAATGCCATGT (reverse); mTNF-α, AACTCCAGGCGGTGCCTATG (forward) and TCCAGCTGCTCCTCCACTTG (reverse); mIFN-γ, TCAAGTGGCATAGATGTGGAAGAA (forward) and TGGCTCTGCAGGATTTTCATG (reverse); mIL-17, CAGGGAGAGCTTCATCTGTGT (forward) and GCTGAGCTTTGAGGGATGAT (reverse); mIL-13, GCAGCATGGTATGGAGTGTG (forward) and TGGCGAAACAGTTGCTTTGT (reverse). The amplification protocol consisted of an initial denaturation step at 95 °C for 5 min, then 35 cycles at 94 °C for 15 s, 60 °C for 15 s, and 72 °C for 15 s, followed by a final extension step at 72 °C for 10 min. The expression level of the above genes was normalized to that of GAPDH.

### Western blot analysis

Whole-cell lysates were prepared in cell lysis buffer (Cell Signaling Technology, Beverly, MA, USA) containing a protease inhibitor cocktail (Tech & Innovation^TM^, Bucheon, Korea Bucheon, Korea) and a phosphatase inhibitor cocktail (sc-45065; Santa Cruz Biotechnology, Santa Cruz, CA). Proteins were then separated on an SDS-PAGE gel and analyzed with the following primary antibodies: PPARα (ac24509; Abcam, Cambridge, MA), p-NF-κB (ab86299; Abcam), NF-κB (sc-372; Santa Cruz Biotechnology), p-IκB-α (sc-101713; Santa Cruz), IκB (sc-371; Santa Cruz), iNOS (PA1-036; Thermo Scientific, Rockford, IL), and β-actin (A5441; Sigma-Aldrich).

### *Aspergillus fumigatus* (*Af*)-induced AD model

BALB/c 8 weeks mice were purchased from Orient Bio Inc. (Seongnam, South Korea). The mice were anesthetized with 60 mg/kg of Zoletil 50® (Virbac, Laboratories, Carros, France), and their dorsal hair was shaved with clippers. 40 µg of *Aspergillus fumigatus* (*Af*) extract (XPM3D3A25; Greer Laboratories, Lenoir, NC, USA) was then epicutaneously applied to a 1 cm^2^ area on the dorsal surface for 5 days. The procedure was repeated twice at 2 week intervals. Control mice were treated similarly with the same amount of PBS alone. The mice (n ≥ 6 animals per group) were then subcutaneously injected with either MSCs (2 × 10^6^ cells/mouse) or MSC-Ex (300 μg/mouse) on day 23 after commencing the *Af* treatment.

### Clinical symptom scoring and epidermal permeability barrier function assessment

The severity of dermatitis was assessed weekly using the following scoring procedure: 0, no symptoms; 1, mild; 2, moderate; and 3, severe. These scores were applied to itching, edema, hemorrhage, excoriation/erosion, and scaling/dryness, expressed as the sum of the scores for these five symptoms (maximum score, 15)^28^. The epidermal permeability barrier function was evaluated by transepidermal water loss (TEWL) measurements with a Vapometer®SWL-3 (Delfin Technologies Ltd., Kuopio, Finland) at the same site and on the same day as the clinical score determination.

### Lymph node cultures

Skin lymph nodes (LNs) were dissected immediately after sacrifice and kept on ice in RPMI-1640 media (Gibco) with 10% fetal bovine serum and 1% penicillin/streptomycin (Gibco). Cell suspensions were obtained by pressing the LNs through a cell strainer (40 µm) (SPL Life science, Pocheon, South Korea) and seeded at 4 × 10^6^ cells/well. LN cells were cultured in the presence of CD3 (3 µg/mL) and CD28 (1 µg/mL) antibodies at 37 °C for 2 days and their supernatants were collected and quantitatively analyzed for mIFN-γ (88-7314-22; Invitrogen), and mIL-17 (88-7371-22; Invitrogen), mIL-4 (KET7007; Abbkine, Wuhan, China) and mIL-10 (900K53EK; PEPROTECH) by enzyme-linked immunosorbent assay (ELISA).

### Total serum immunoglobulin E (IgE) measurement

Blood samples were obtained from the inferior vena cavas of the mice immediately after sacrifice. Serum was separated from the blood clots by centrifugation at 2,000 rpm for 10 minutes at 20 °C and stored at −80 °C. Total serum immunoglobulin E (IgE) concentrations were measured by ELISA (#555248; BD).

### Histological evaluation

Dermatitis severity was assessed microscopically in the mice as indicated in Supplementary Table 1 using the following scoring system: skin sections of the dorsal surface were stained with hematoxylin and eosin (H&E) and graded for inflammation (0, none; 1, mild; 2, moderate; 3, severe), depth of fibrosis (0, none; 1, ~0.3 mm; 2, ~0.5 mm; 3, ~0.8 mm; 4, ~1 mm), parakeratosis and/or hyperkeratosis (0, none; 1, reduced; 2, no change, hypergranulosis (0, none; 1, reduced; 2, no change) and psoriasiform epidermal hyperplasia (0, none; 1, reduced; 2, no change). The score for each parameter was multiplied by a factor reflecting the percentage of tissue involvement to yield the final score.

### Statistical analysis

All data are expressed as the mean ± s.d. Statistical significance was evaluated using the non-parametric Wilcoxon rank sum test and defined as P < 0.05.

## Supplementary information

Supplementary figures
