# Supplementary material for: Umbilical cord-derived mesenchymal stem cell extracts ameliorate atopic dermatitis in mice by reducing the T cell responses
Source: Sci Rep. 2019 Apr 29;9:6623. doi: 10.1038/s41598-019-42964-7 (PMC6488580; doi:10.1038/s41598-019-42964-7)
Supplement: Supplementary file 1 — Supplementary figures [file 41598_2019_42964_MOESM1_ESM.pdf]

Umbilical cord-derived mesenchymal stem cell extracts ameliorate atopic dermatitis in mice by reducing the T cell responses

Ji-young Song[1], Hyo Jeong Kang[2], Hyun Min Ju[1], Arum Park[1], Hyojung Park[1], Joon Seok Hong[3], Chong Jai Kim[2], Jae-Yoon Shim[4], Jinho Yu[5] & Jene Choi[2]*

[1]Institute for Life Science, University of Ulsan College of Medicine, Asan Medical Center, Seoul, Korea, [2]Department of Pathology, University of Ulsan College of Medicine, Asan Medical Center, Seoul, Korea, [3]Department of Obstetrics and Gynecology, Seoul National University Bundang Hospital, Gyeonggi-do, Korea, [4]Department Obstetrics and Gynecology, [5]Department of Pediatrics, Asan Medical Center, University of Ulsan College of Medicine, Seoul, Korea.

*Correspondence:

Jene Choi, PhD, Department of Pathology, University of Ulsan College of Medicine, Asan Medical Center, 88 Olympic-ro 43-gil, Songpa-gu, Seoul 05505, Korea.

Telephone: +82-2-3010-4555; Fax: +82-2-472-7898; E-mail: jenec@amc.seoul.kr

Jinho Yu, MD, PhD, Department of Pediatrics, University of Ulsan College of Medicine, Asan Medical Center, 88 Olympic-ro 43-gil, Songpa-gu, Seoul 05505, Korea.

Telephone: +82-2-3010-3922, Fax: +82-2-473-3725, E-mail: jinhoyu@amc.seoul.kr

Acknowledgments

This work was supported by grants from the Korea Health Technology R&D Project through the Korea Health Industry Development Institute (KHIDI), Ministry of Health, Welfare and Family Affairs, Republic of Korea (HI15C2111), from the National Research Foundation of

Korea (NRF-2017R1A2B4002758), and from the Asan Institute for Life Sciences, Asan Medical Center, Seoul, Korea (W16-644).

Author contributions

Ji-young Song, acquisition of data; Hyo Jeong Kang, review of pathology; Hyun Min Ju, acquisition of data; Arum Park, technical support; Hyojung Park, technical support; Joon Seok Hong, obtainment of study materials; Chong Jai Kim, analysis and interpretation of data; Jae-Yoon Shim, obtainment of study materials; Jinho Yu, conceived and designed the study; Jene Choi, supervised the study, obtaining funding and wrote the manuscript.

Additional information

The authors declare no conflicts of interest.

Supplementary Table 1. Histological scoring system for atopic dermatitis severity

**Score**	**Variables**
	Inflammation
0	None
1	Mild
2	Moderate
3	Severe
	Depth of fibrosis
0	None
1	< 0.3 mm
2	0.3 ~ ≤ 0.5 mm
3	0.5 ~ ≤ 0.8 mm
4	0.8 ~ 1 mm
	Parakeratosis or/and hyperkeratosis
0	None
1	Reduced
2	No change
	Hypergranulosis
0	None
1	Reduced
2	No change
	Psoriasiform epidermal hyperplasia
0	None
1	Reduced
2	No change

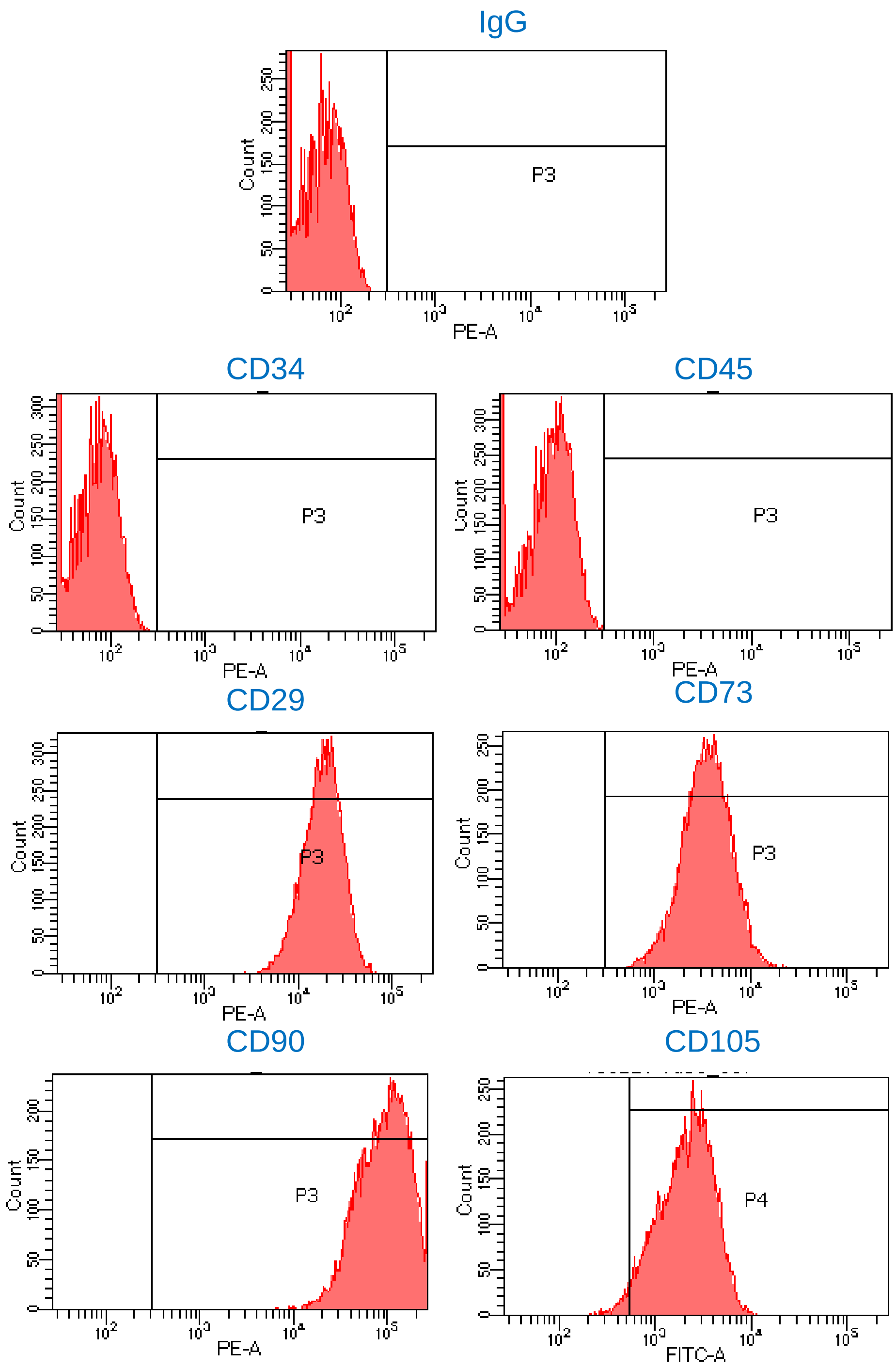

Figure S1. UC-MSCs were characterized with a panel of surface markers using flow cytometric analyses. UC-MSCs were positive for CD29, CD73, CD90 and CD105 and were negative for CD34 and CD45.

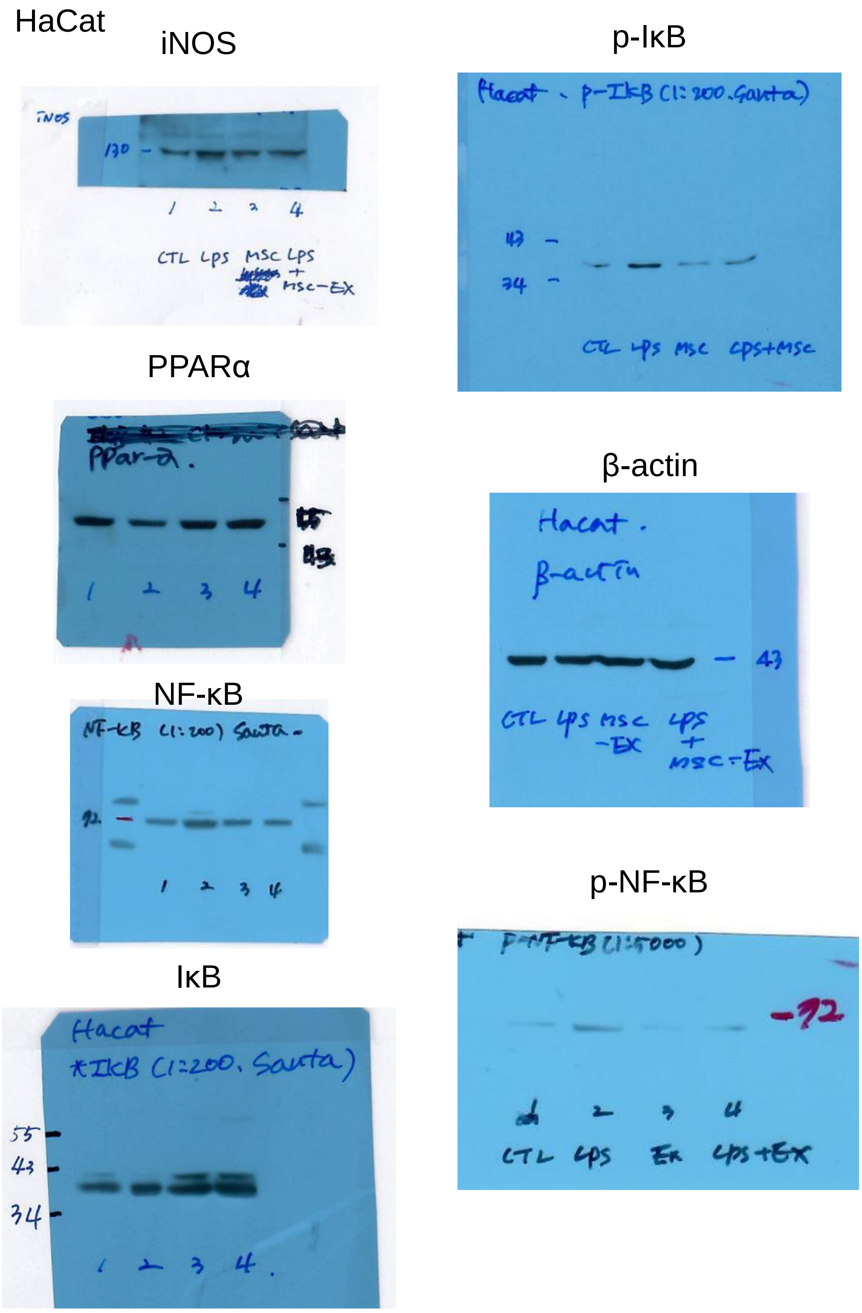

Figure S2. Western original data

PPARα

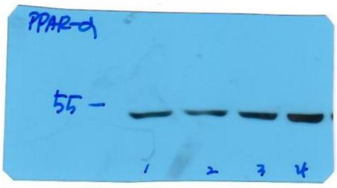

β-actin

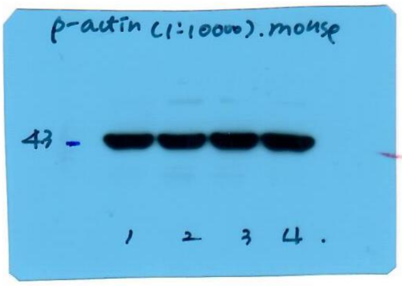

NF-κB

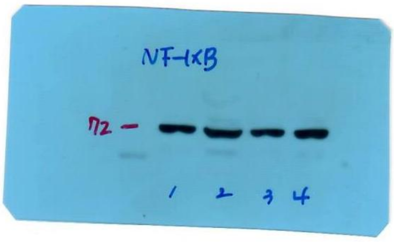

p-NF-κB

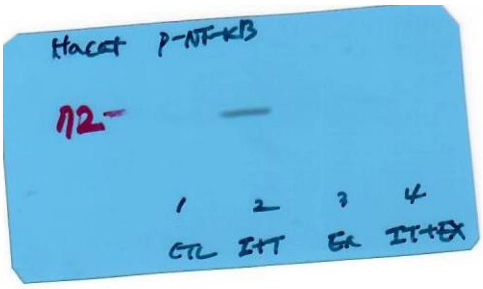

p-IκB

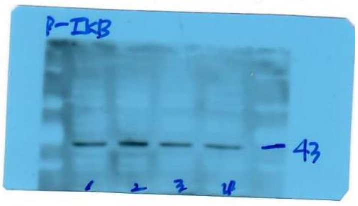

IκB

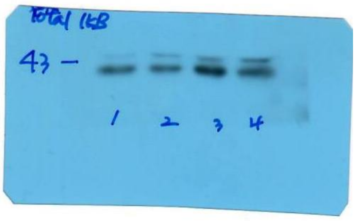

iNOS

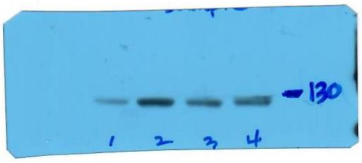

Figure S2. Western original data

Mouse skin

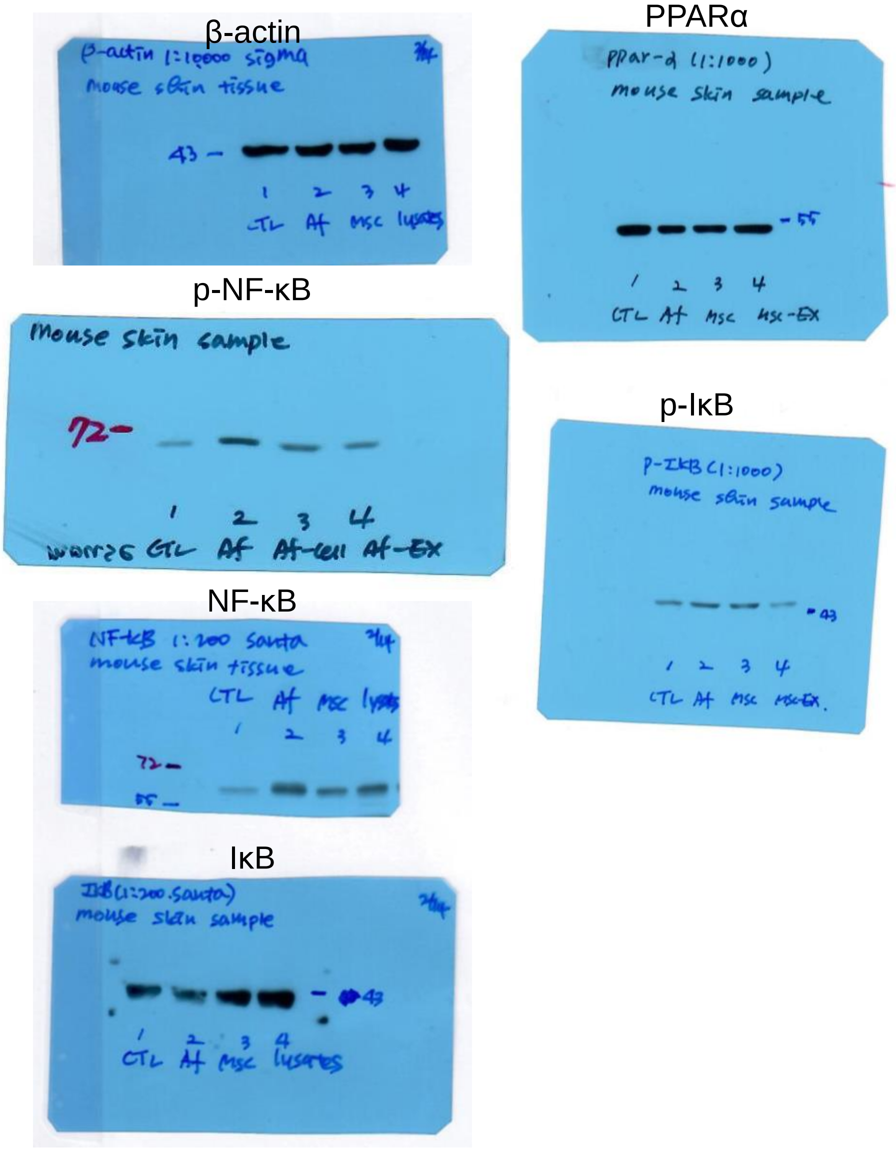

Figure S2. Western original data
